# Comparative transcriptomics of genetically divergent lines of chickens in response to Marek’s disease virus challenge at cytolytic phase

**DOI:** 10.1371/journal.pone.0178923

**Published:** 2017-06-07

**Authors:** Kunzhe Dong, Shuang Chang, Qingmei Xie, Alexis Black-Pyrkosz, Huanmin Zhang

**Affiliations:** 1 USDA, Agricultural Research Service, Avian Disease and Oncology Laboratory, East Lansing, Michigan, United States of America; 2 ORISE Fellow, USDA, Agriculture Research Service, Avian Disease and Oncology Laboratory, East Lansing, Michigan, United States of America; 3 College of Veterinary Medicine, Shandong Agricultural University, Tai’an, Shandong, China; 4 College of Animal Science, South China Agricultural University, Guangzhou, China; Sun Yat-Sen University, CHINA

## Abstract

Marek’s disease (MD), caused by Marek’s disease virus (MDV), remains an economically significant threat to the poultry industry worldwide. Genetic resistance to MD is a promising alternative strategy to augment current control measures (vaccination and management). However, only a few functional genes reportedly conferring MD resistance have been identified. Here, we performed a comparative transcriptomics analysis of two highly inbred yet genetically divergent lines of chickens (line 6_3_ and 7_2_) that are resistant and susceptible to MD, respectively, in response to a very virulent plus strain of MDV (vv+MDV) challenge at cytolytic phase. A total of 203 DEGs in response to MDV challenge were identified in the two lines. Of these, 96 DEGs were in common for both lines, in addition to 36 and 71 DEGs that were specific for line 6_3_ and 7_2_, respectively. Functional enrichment analysis results showed the DEGs were significantly enriched in GO terms and pathways associated with immune response. Especially, the four DEGs, *FGA*, *ALB*, *FN1*, and *F13A1* that reportedly facilitate virus invasion or immunosuppression, were found to be significantly up-regulated in the susceptible line 7_2_ but down-regulated in the resistant line 6_3_ birds. These results provide new resources for future studies to further elucidate the genetic mechanism conferring MD resistance.

## Introduction

Marek’s disease (MD), caused by Marek’s disease virus (MDV), is a serious lymphoproliferative disease of chickens, which is roughly estimated to incur an annual loss of $2 billion for the world poultry industry, primarily resulting from MD related mortality, reduced egg production, and meat condemnations [1]. Although commercially available vaccines have been used to protect chickens from MD since the 1970s [2], MD still remains a serious threat due to increasing MD outbreaks resulting from emergence of more virulent strains of MDV combined with incomplete immunity that is elicited by vaccination alone [3]. Therefore, alternative strategies to augment existing vaccine control measures are greatly needed.

Genetic resistance has recently proved to be an attractive strategy as it is more predictable, economically feasible, free of negative environment impact, and takes full advantage of state of the art techniques in modern genetics and genomics research [4,5]. A dramatic example is that chickens with a major histocompatibility complex (MHC) B*19 haplotype (line P) incurred 100% MD tumor incidence, whereas chickens with a B*21 haplotype (line N) were observed with a 55% tumor incidence 30 days post infection (DPI) with a very virulent strain of MDV [6]. Genetic resistance to MD attributable to MHC *B*-haplotypes in chicken is well documented [7,8]. Furthermore, there are lines of chickens that share common *B*-haplotypes but differ in MD resistance/susceptibility, which suggests there are other genes that confer genetic resistance to MD in addition to MHC genes. One of the most notable examples is the highly-inbred lines 6_3_ and 7_2_, developed and maintained at the USDA, Agriculture Research Service, Avian Disease and Oncology Laboratory (ADOL) at East Lansing, Michigan, U.S.A. Both lines 6_3_ and 7_2_ are homozygous for B*2 haplotype. However, the line 6_3_ is highly resistant to MD, but the line 7_2_ is highly susceptible [9].

In the past decades, considerable effort has been put forward to explore the fundamental basis underlying genetic resistance to MD. Such effort includes, for instance, quantitative trait locus (QTL) mapping [10–14], which was successful in identifying a series of loci or domains of the chicken genome associated with genetic resistance or susceptibility to MD, and immune-specific genes or whole-genome microarray analyses [4,15–24], which identified genes with dysregulated expression in response to MDV challenge in embryo fibroblasts, peripheral blood leukocytes, lymphoid organs or epithelial tissues of chickens. These studies have identified candidate genes involved in a wide range of biological processes involved with host-pathogen interactions, such as antigen presentation, interferon (IFN) response, signal transduction, cytoskeleton, immune response, cell surface molecules and cell apoptosis. Microarray as a gene expression analysis platform, however, has some limitations, including discrepancy in hybridization efficiency, cross-hybridization background between array probes, reliance on existing knowledge and genome sequences, and a dynamic range of detection on account of both background and saturation of signals [25,26].

Recently, RNA sequencing (RNA-Seq) coupled with next-generation sequencing (NGS) technologies has rapidly become a new standard protocol for global profiling of transcriptomes in most. Several studies have employed RNA-Seq to explore gene expression profiles of chickens with MD [27–29]. Despite the extensive efforts, molecular mechanisms of genetic resistance to MD, however, remain far from fully elucidated. This study took the advantage of using two highly inbred and genetically divergent lines (6_3_ and 7_2_) of chickens to advance the insight of genetic resistance to MD by comparatively exploring the transcriptomes and identifying differentially expressed genes (DEGs) in response to a very virulent plus strain of MDV (vv+MDV) challenge by NGS at the cytolytic phase.

## Materials and methods

### Ethics statement

All chickens used in this study were housed in a BSL-2 experimental facility under controlled conditions during the trial. Feed and water were supplied *ad libitum*. The chickens were observed daily throughout the entire duration of the experiment. The animal challenge experiment was approved by USDA, Agriculture Research Service, Avian Disease and Oncology Laboratory Institutional Animal Care and Use Committee (IACUC). The ADOL IACUC guidelines (April 2005) and the Guide for the care and use of Laboratory Animals by Institute for Laboratory Animal Research (2011) were closely followed throughout the experiment.

### Experimental animals and design

White Leghorn chickens from two highly inbred lines, lines 6_3_ and line 7_2_, which were developed and maintained at the USDA, Agriculture Research Service, Avian Disease and Oncology Laboratory, East Lansing, Michigan, U.S.A. [9], were used in this study. Line 6_3_ is known to be relatively resistant to MD, while line 7_2_ is highly susceptible. Chickens sampled from each line were divided into two groups on the day of hatch, one as MDV challenge group, the other as control group. Each of the chicks in the MDV challenge groups of both lines was inoculated intraabdominally with 500 plaque-forming units of 648A passage 10 MDV on day 5 post hatch. No inoculation was implemented to chickens of the control groups. On the 5^th^ day post infection, three chicks from each of the treatment groups were randomly euthanized following ADOL IACUC’s CO_2_ gas euthanasia protocol (ADOL IACUC SOP #11). Spleen samples from the chicks were individually collected, immediately placed into RNAlater solution (Qiagen, Valencia, CA, USA), and stored at -20°C until RNA extraction.

### RNA extraction and sequencing

Total RNA was extracted using TRIzol reagent (Invitrogen, Carlsbad, CA, USA) following the manufacturer’s instructions. RNA concentration and quality were determined using an Agilent 2100 Bioanalyzer (Agilent Technologies, Santa Clara, CA, USA). Equal amounts of RNA samples from three biological replicates within each line for each treatment group were pooled in preparation to construct standard cDNA libraries using Illumina TruSeq kits and reagents following the manufacturer’s protocol for deep sequencing. The libraries were sequenced on an Illumina HiSeq2000 sequencer for single end 50 base sequencing run. The post sequencing processes, including image analysis, base calling, and Q-Score calculation, were carried out using Real Time Analysis (v1.13.48); read demultiplexing and conversion to final FASTQ files, using CASAVA (v1.8.2) software tools (Illumina Inc., San Diego, CA, USA). The library preparation, RNA sequence read extraction, and preliminary read quality control were performed at the Research Technology Support Facility, Michigan State University.

### Mapping and gene expression quantitation

Sequence adaptors were first removed in the first quality control process using Trimmomatic (version 0.32) software [30] to obtain the past filter (PF) reads. Low quality bases were further trimmed from the PF reads using custom Python scripts eliminating the first 15 nucleotides. Sickle (v1.33) [31] was used with a sliding window average quality score of 30, removal of reads with “N”s, and a minimum read length of 30 bps, to produce the final set of high quality reads. The high quality reads were then mapped to the chicken reference genome (galGal4) using TopHat2 (v2.0.12) [32] and Bowtie2 (v2.2.3) [33] with default parameters. Transcript abundance and differential expression of genes were estimated with Cufflinks (v2.2.1) [34]. Per kilobase of transcript per million (FPKM) values were obtained to quantify relative expression of transcripts and all calculations using FPKM were performed on log10(FPKM+1) transformed expression data.

### Analyses for differentially expressed genes

Transcripts with a FPKM value >0.3, an optimized threshold providing the best balance between false positive discovery and negative gene expression proposed by Ramskold, et al. [35], in at least one of two groups under each comparison were included in the analysis. In each of the pairwise comparisons, DEGs were first identified by using the DESeq R package [36] with raw reads count of each gene, which was calculated by the HTSeq software [37], and was then filtered following a criteria of a false discovery rate (FDR) <0.05 and absolute Log2 Fold Change (FC) > 1. To better understand the functional involvements of these DEGs, g:Profiler (http://biit.cs.ut.ee/gprofiler/index.cgi) [38] was used for the gene annotation, GO and pathway enrichment analysis.

### Droplet Digital^TM^ PCR validation of gene expression

To spottily validate the expression of genes determined by RNA-Seq, three genes from each of the treatment groups were selected and re-evaluated on a Droplet Digital^TM^ PCR (QX200^TM^ ddPCR system; Bio-Rad Laboratories, Inc., Hercules, CA, USA). Genes that were selected for ddPCR re-evaluation included both significant DEGs, non-DEGs, and up- as well as down-regulated genes (**S1 Table**). A total of seven genes was selected. The ddPCR primers for each of the selected genes were designed with Primer3Plus (http://www.bioinformatics.nl/cgi-bin/primer3plus/primer3plus.cgi/), and are listed in **S2 Table**. The cDNA samples used in ddPCR validation were reversely transcribed from individual RNA samples (the same samples pooled in preparation of the standard cDNA libraries for RNA-Seq) using the iScript^TM^ RT Supermix Kit (Cat No. 170–8841) and following the manufacturer’s instructions (Rio-Rad). A ddPCR reaction mixture of 25 μL in final volume was initially prepared per gene per biological sample including 2 μL of cDNA, 12.5 μL of EvaGreen Supermix (Cat No. 1864034), 0.5 μL of each of the forward and reverse primers (200 nM; synthesized by Eurofins Genomics, Huntsville, AL), and 9.5 μL of nuclease-free water. Of these, 20 μL were loaded into one of 8 sample channels of a DG8^TM^ cartridge (Cat No. 1864008, Bio-Rad). Each oil well was loaded with 70 μL of droplet generating oil (Cat No. 1864006, Bio-Rad). The loaded DG8^TM^ cartridges were placed on a QX200^TM^ droplet generator (Bio-Rad) to generate the digital droplets. Forty μL of the generated droplet emulsion for each sample were transferred to a well in a 96-well PCR plate followed by polymerase chain reaction with EvaGreen on a C1000^TM^ Thermal Cycler (Bio-Rad). The cycling conditions were 95°C for 5 min, followed by 40 cycles of 95°C for 15 s, 58°C for 60 s, and a final extension step of 98°C for 10 min. The droplets post PCR were read well by well on a QX200^TM^ droplet reader (Bio-Rad). PCR-positive and PCR-negative droplets in each of the wells were counted and analyzed with the QuantaSoft software (version 1.7, Bio-Rad).

## Results

### Statistical assessments of the observed global gene expression profiles

A total of 32.4, 33.0, 40.4, and 28.3 million past filter (PF) reads were extracted from the RNA-Seq data for line 6_3_ control (L6_3_Cont), line 7_2_ control (L7_2_Cont), line 6_3_ MDV-challenged (L6_3_MDV) and line 7_2_ MDV-challenged (L7_2_MDV) treatment groups, respectively. Approximately 81.9–82.3% of the PF reads remained after trimming of low quality bases. About 85.6–88.1% of the high-quality reads were successfully mapped to the chicken reference genome (**S3 Table**). With an expression threshold of fragments FPKM >0.3, a total of 13,105 genes were identified in the spleen tissue samples of the control and MDV-challenged groups of both lines, with a range of 10,956 to 11,368 genes among the treatment groups (**S1A Fig**). At a global view, the expression levels of over 4000 genes per group were relatively low (FPKM <10), and over 7000 genes were relatively high (10≤ FPKM <500). The expression levels of the top 200 plus genes were at or above 500 FPKM value (**S1B Fig**). No significant difference was observed between the average overall gene expression levels of the treatment and line groups (**S1C Fig**).

Principal component analysis (PCA) clearly depicted the differences in variability of gene expression between control (L6_3_Cont and L7_2_Cont) and MDV infected (L6_3_MDV and L7_2_MDV) groups (PC1, 49.8%), as well as between the two lines (PC2, 28.2%) (**S2A Fig**). A hierarchical cluster analysis of the transcriptomes showed the difference in expression level was measurably greater between the MDV-challenged and control groups than between the lines (**S2B Fig**).

### Differentially expressed genes induced by MDV

**Fig 1:**
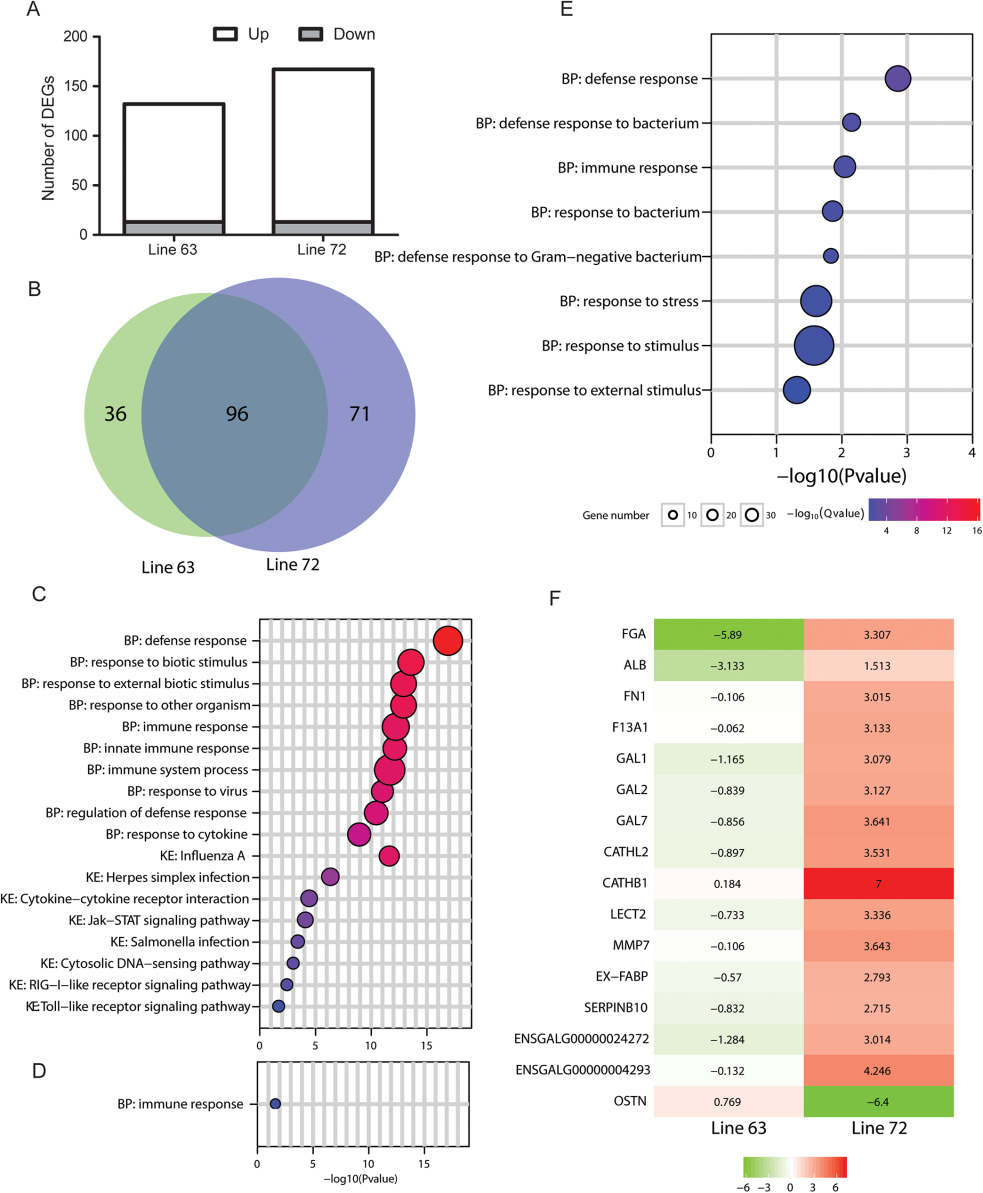
Differentially expressed genes (DEGs) of line 6_3_ and 7_2_ chickens in response to MDV challenge. (A) Depicted different number of DEGs identified in each of the lines. (B) Venn diagram of DEGs between the lines 6_3_ and 7_2_. (C) Top ten gene ontology (GO) terms and significant pathways with wihch the common DEGs are associated; BP: biological process. KE: KEGG pathway. (D) Significant GO term of line 6_3_-specific DEGs. (E) Significant GO and pathway terms of line 7_2_-specific DEGs. (F) Heatmap showing the top DEGs that were very different in expression between the lines 6_3_ and 7_2_ in response to MDV challenge. The color bar represents the log2 Fold Change (FC) in response to MDV challenge (the FC value for *CATHB1* in line 7_2_ bar was “Inf” and was arbitrarily set to 7 in the chart).

To explore genes potentially conferring MD resistance, DEGs in response to MDV challenge were identified between the MDV-infected and control groups in each line. A total of 132 and 167 DEGs were identified with a FDR < 0.05 and an absolute Log2 FC > 1 in lines 6_3_ and 7_2_, respectively (see **S4** and **S5 Tables** for the full lists of DEGs). In both lines, most of the DEGs (90.15% in line 6_3_ and 92.22% in line 7_2_) were up-regulated in response to MDV challenge (**Fig 1A**).

Of the DEGs, there were a total of 96 DEGs presented in both lines, while 36 and 71 DEGs were unique in line 6_3_ and line 7_2_, respectively (**Fig 1B**). All of the DEGs in common to both lines, except *FGA*, were consistent in change direction in both of the lines.

To better understand potential biological events that the DEGs were involved with, a gene enrichment analysis was conducted. The 96 common DEGs were significantly (P <0.05) enriched in 125 Gene Ontology (GO) terms of biological processes and 9 KEGG pathways (**S6 Table**). Notably, the top significant GO terms and all of the key pathways were involved in immune response (**Fig 1C**), which included defense response, innate immune response, response to virus, cytokine-cytokine receptor interaction pathway, Jak-STAT signaling pathway and RIG-I-like receptor signaling pathway.

The 36 line 6_3_ specific DEGs were significantly enriched in one GO term, the immune response (**Fig 1D**). The 71 line 7_2_ specific DEGs were enriched in the same GO term in addition of 7 other major GO terms predominantly related to defense response, response to stress, and response to stimulus (**Fig 1E**). Arginine biosynthesis pathway was also identified with statistical significance for the DEGs unique for the line 7_2_.

There was a total of 16 DEGs (*FGA*, *ALB*, *FN1*, *F13A1*, *GAL1*, *GAL2*, *GAL7*, *CATHL2*, *CATHB1*, *LECT2*, *MMP7*, *EX-FABP*, *SERPINB10*, *ENSGALG00000004293*, *ENSGALG00000024272* and *OSTN*) that were either highly up- or down-regulated in expression in the line 7_2_ with |Log2 FC| ≥ 2.7 while were up or down-regulated in the line 6_3_ with |Log2 FC| ≤ 1.3 or were significantly up-regulated in the line 7_2_ with Log FC > 1.5 and significantly down-regulated in the line 6_3_ with Log2 FC < -3.1 (*FGA* and *ALB*) in response to MDV challenge (**Fig 1F**). The DEGs were considered the top DEGs that were very different in expression between the two genetic lines of chickens in response to MDV challenge.

### Differentially expressed genes between lines 6_3_ and 7_2_

**Fig 2:**
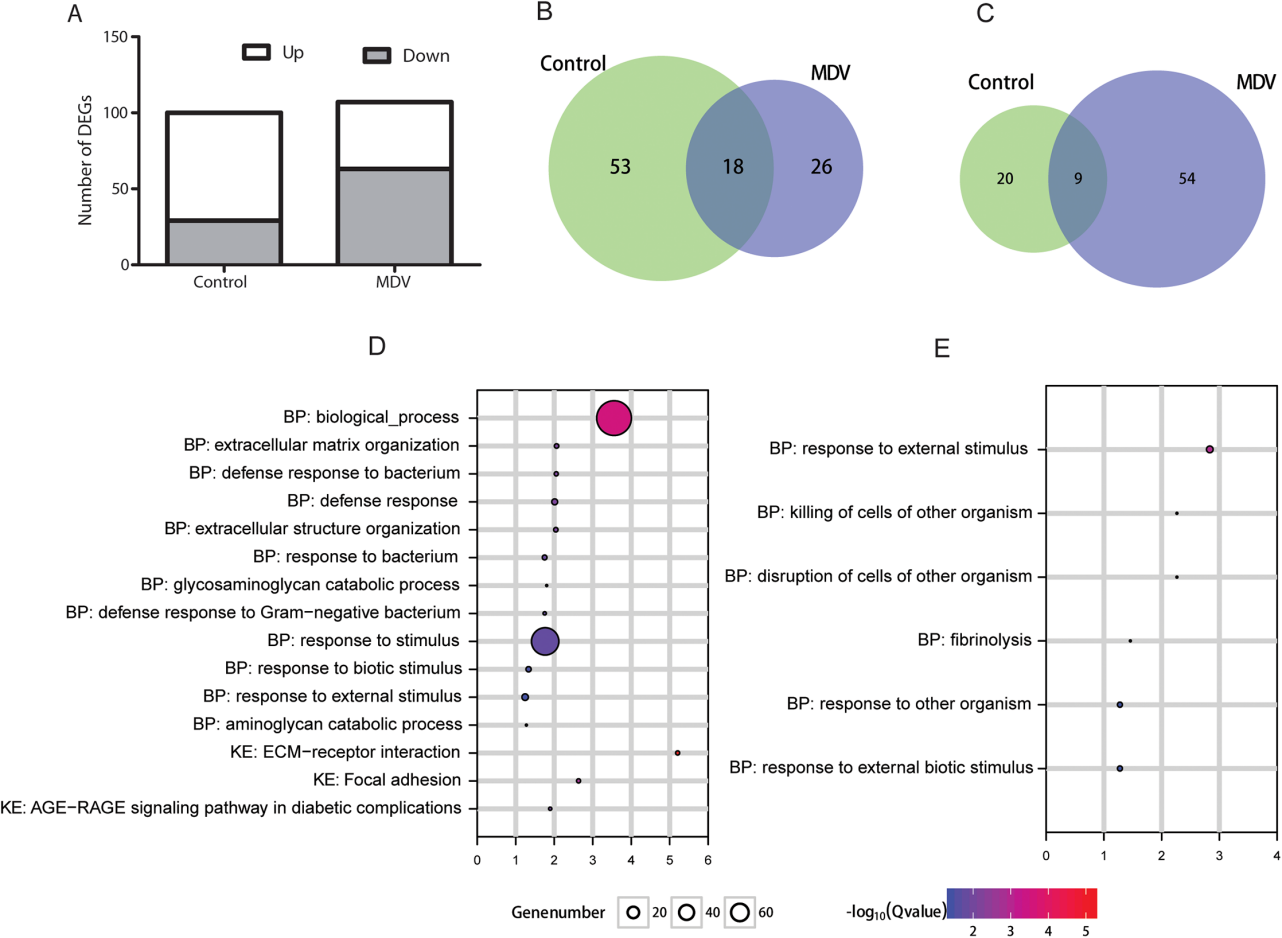
Differentially expressed genes (DEGs) between the two lines. (A) Summary of the number of DEGs between the two lines for each treatment. (B) Venn diagram showing the number of up-regulated DEGs in line 6_3_ relative to line 7_2_ for different treatments. (C) Venn diagram of number of up-regulated DEGs in line 7_2_ relative to line 6_3_ for different treatments. (D) Significant GO terms of DEGs between the two lines in control birds. BP: biological process. KE: KEGG pathway. (E) Significant GO terms of DEGs between the two lines in MDV-infected birds BP: biological process.

A total of 100 and 107 genes were identified, which were differentially expressed between line 6_3_ and line 7_2_ (Line 6_3_/Line 7_2)_ chickens without (control) and with MDV challenge, respectively (FDR < 0.05 and an absolute Log2 FC > 1.0; **S7 and S8 Tables**, **Fig 2A**). Of the DEGs that were expressed significantly higher in line 6_3_ than in 7_2_ chickens, 26 genes were observed only in the MDV challenged group, 53 only in the control group, and 18 in both MDV challenged and control groups (**Fig 2B**). In contrast, 54 genes were expressed at significantly lower levels only in the MDV challenged group, 20 in the control group, and 9 genes in both groups (**Fig 2C**).

The 71 (53+18) DEGs with significantly higher expression levels between line 6_3_ and line 7_2_ control birds were significantly enriched in 12 GO terms and 3 pathways (**Fig 2D**). The majority of the GO terms are involved with immune response. In contrast, the 63 (9+54) DEGs significantly expressed at lower levels between line 6_3_ and 7_2_ MDV challenged groups were significantly enriched in 6 major GO categories (**Fig 2E**). No significant GO terms were identified for either the DEGs expressed at higher levels in line 6_3_ MDV challenged or the DEGs expressed at lower levels in line 6_3_ control groups compared to line 7_2_ control group.

### Differential expression patterns validated by ddPCR

**Fig 3:**
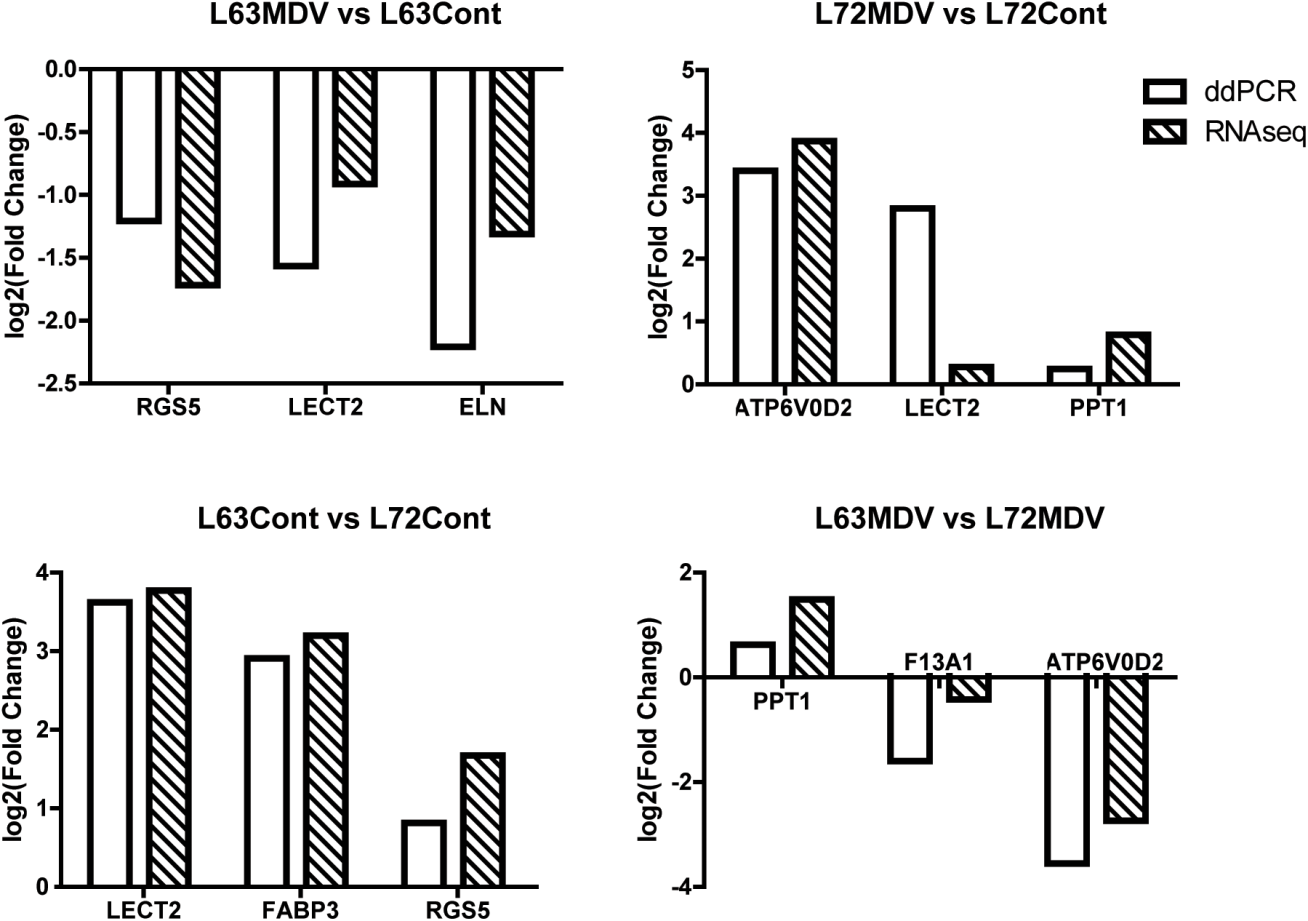
Plotted relative fold changes of selected genes determined by RNA-Seq and droplet digital PCR. The fold changes are expressed as the ratio of L63MDV/L63C, L72MDV/L72C, L63C/L72C, L63MDV/L72MDV, respectively.

Although the birds used in this study within each of the lines are highly inbred (homozygosity is > 99%), and RNA-seq of pooled samples within treatment has been commonly used in transcriptome studies [39,40], there are concerns remained to be aware of on detected differential expression between treatment groups due to loss of potential biological variation within each line of birds. To validate the gene expression patterns detected by RNA-Seq data, three genes from each of the pairwise comparison groups were selected and re-evaluated for expression pattern using individual RNA samples on a droplet digital PCR (ddPCR) platform [41], which is a highly precise and absolute nucleic acid quantification system. The ddPCR data confirmed the relative expression patterns of the selected genes identified by the RNA-Seq data (**Fig 3**).

## Discussion

MDV infection constitutes a complex process that evokes various components of the host immune system coupled with virus-induced physiological changes, and eventually leads to lymphoid neoplasms in susceptible chickens [42–45]. Dissecting global transcriptome of profiles of genetically divergent lines of chickens and difference in transcriptomic changes in response to MDV challenge between varied genetic lines of birds could lead to critical insights into the genetic mechanism that confers MD resistance. Any advancement in such basic knowledge is of great interest to the poultry industry and to the field of cancer biology. In this study, we took advantage of RNA-Seq technology and the inbred chicken lines 6_3_ and 7_2_, to expand current candidate gene list and to extend the fundamental understanding of genetic mechanism underlying MD and MD resistance. Whole-transcriptome analysis of the two highly inbred lines of chickens that significantly differ in MD resistance was conducted with splenic total RNA samples taken at 5 DPI, the early cytolytic phase.

A thorough but non-exhaustive literature search showed over 5,000 genes were reported from RNA-Seq and/or quantitative real-time PCR studies, which were dysregulated in expression under the induction of MDV challenge at varied stages [15,17,19,20,22–24,27,28,46–48]. In this study, a total of 203 genes were identified in the two genetic lines of chickens differentially expressed in response to MDV challenge under the controlled conditions at 5 DPI. Of these, 153 genes had been reported in one or up to 7 studies, and the other 50 genes are reported here for the first time (**S9 Table**).

It is clearly noticeable that more genes (26.5%) were dysregulated in expression in the susceptible line 7_2_ birds than those of resistant line 6_3_ in response to MDV challenge (**Fig 1A**). A similar trend was observed between genetically divergent strains of mice in response to influenza A virus infection [49]. This phenomenon might indicate either that disease susceptibility is positively associated with the number of genes that are hypersensitive to pathogen induction or higher viral titer in susceptible birds, in contrast to the resistant ones, induces more genes, especially immune genes, to undergo dysregulated expression [50].

Of the significantly dysregulated genes, 96 were common to both lines of chickens (**Fig 1B**). The majority of those genes were associated with GO categories of immune responses (**Fig 1C**), and were involved with key pathways including RIG-I-like receptor signaling pathway, Toll-like receptor signaling pathway and NOD-like receptor signaling pathway, which is associated with pathogen recognition by innate immune system [51,52], Cytokine-cytokine receptor interaction and Jak-STAT signaling pathway, which are engaged in critical biological processes including both innate and adaptive inflammatory host defenses, cell growth, differentiation and death, angiogenesis and repair processes in restoration of homeostasis [53,54]. These DEGs and pathways, however, are unlikely to underlie the known difference in genetic resistance to MD between the two lines of chickens.

Although all chickens are susceptible to MDV infection, there are significant differences in MD incidence and survival day post MDV challenge between the two lines of chickens [55,56]. Thus, the line-specific DEGs may constitute partial genetic basis underlying the observable phenotypic difference in MD incidence and survival day [44,57]. We identified 36 and 71 line-specific DEGs in the MD resistant line 6_3_ and susceptible line 7_2_ chickens, respectively (**Fig 1B**). The 36 line-specific DEGs of line 6_3_ are significantly associated with a single major GO category: immune response, while the 71 DEGs of line 7_2_ are significantly associated 7 other major GO categories, in addition to the immune response GO term.

DEGs in response to MDV challenge could modulate resistance or susceptibility to MD. On one hand, some DEGs might be triggered by the host immune system to recognize and eliminate invading pathogens; on the other, DEGs might be exploited by the virus to facilitate its infection processes [58–60]. All of the 16 top DEGs, except *ALB* and *OSTN*, were significantly up-regulated in the susceptible line 7_2_ birds in response to MDV challenge, suggesting that the majority of these DEGs, if not all of them, are bound to fail in preventing host susceptibility to MD (**Fig 1F**) since the line 7_2_ birds are highly susceptible to MD [9,61]. Searched literatures show the 16 DEGs, except *OSTN*, are functionally more or less involved with immune response. Eleven of the 16 genes potentially enhance host immune response, which include *GAL1*, *GAL2*, *GAL7*, *CATHL2*, *CATHB1*, *LECT2*, *MMP7*, *SERPINB10*, *EX-FABP*, *ENSGALG00000004293* and *ENSGALG00000024272*. Especially, the three gallinacin (*GAL1*, *GAL2* and *GAL7*) and the two cathelicidin (*CATHL2* and *CATHB1*) genes are reportedly to contribute to antivirus activity and are key components of the innate immune system [62,63]. LECT2 (Leukocyte cell-derived chemotaxin 2) is a multifunctional protein characteristically similar to cytokines. Earlier studies showed *LECT2* improved protective immunity in bacterial sepsis [64]. Lack of LECT2 (*LECT2* knock-out) in mice resulted in more serious hepatitis induced by concanavalin A (in contrast to wild type mice) [65]. The *MMP7* is a member of the family of matrix-degrading metalloproteinases (MMPs) that are essential to tissue remodeling. MMP7^-/-^ mice were found to be more susceptible to PR/8 influenza infection [66]. *SERPINB10*, encoding a predominantly intracellular serpin, was reported to be involved in immunity [67]. *EX-FABP* is reportedly associated with inflammatory and antibacterial defense [68,69]. The other two genes, *ENSGALG00000004293* and *ENSGALG00000024272*, may play important roles in response to stimulus based on g:Profiler [38] annotation., Given the widely involvement in immune functions, these DEGs, intuitively, should contribute to genetic resistance, rather than susceptibility, to MD. Yet, the line 7_2_ birds are known highly susceptible to MD, suggesting that the collective effects of these DEGs are insufficient to prevent MDV-induced tumorigenesis. Furthermore, should these immunity related DEGs in any way facilitate MDV infection processes in the line 7_2_ birds, which consequently leads to the high susceptibility phenotype of the line [60,70]? Further functional studies of the DEGs are warranted to determine the exact biological roles these DEGs play in the event of MDV infection and tumor formation.

Four of the 16 top DEGs are known to play a role in facilitating virus invasion. *FGA* encodes fibrinogen alpha chain that is of strong immunosuppressive activities [71] and an essential mediator during the initial phase of bacterial invasion [72,73]. *FGA* was significantly down-regulated in the resistant line 6_3_ (FC = -5.89), and up-regulated in the susceptible line 7_2_ (FC = 3.31). *ALB* encodes monomeric protein that primarily acts as a carrier for steroids, fatty acids, and thyroid hormones and functionally stabilizes extracellular fluid volume. Overexpression of *ALB* in ducks led to down-regulated expression of *INF-β* and *Mx1* genes [74], both of which are antivirus genes [75,76]. *ALB* was significantly down-regulated in line 6_3_ (FC = -3.13). *FN1* is associated with actin polymerization, which has been shown to promote viral binding and entry into target cells [77]. *FN1* was also reportedly to facilitate viral infection through interaction with virus, such as HIV-1, in activated CD4+ T cells [78]. *FN1* was significantly upregulated in line 7_2_ (FC = 3.02). The last one of the four DEGs was *F13A1*, and reportedly it is essential in the event of influenza virus infection [79]. *F13A1* was significantly up-regulated in expression line 7_2_ (FC = 3.13) in response to MDV challenge. Based on the literatures, these four DEGs functionally ought to be associated with MD susceptibility. Furthermore, the levels of expression of these four DEGs in response to MDV challenge were positively associated with MD susceptibility (line 7_2_ characteristics) and negatively associated with MD resistance (line 6_3_ characteristics). The mechanism that regulates the gene expression level in chicken when it is exposed to MDV deserves immediate attention in future investigation.

## Conclusions

A whole genome transcriptome analysis was carried out employing RNA-Seq analysis followed with ddPCR validation in two genetically divergent inbred lines of White Leghorns, one is resistant to MD and the other is highly susceptible. In response to MDV challenge, 26 genes were significantly upregulated in expression, and 54 genes, downregulated in expression in the MD resistant line in contrast to the highly susceptible line of birds at cytolytic phase. The top four candidate genes, *FGA*, *ALB*, *FN1* and *F13A1*, were either significantly upregulated in the MD susceptible birds in response to MDV challenge or significantly downregulated in MD resistant birds. One of the four genes, the albumin gene (*ALB*), is reported for the first time in response to MDV challenge. The elevated expression of these four genes highly likely conferred susceptibility to MD in line 7_2_ birds, and vice versa, in the line 6_3_ birds. The findings of this study are largely in good agreement with reports of similar studies and provide a rich resource for further studies pursing the genetic mechanisms conferring MD resistance. A word of caution acknowledged here again is that all of the DEGs identified in this study were based on pooled RNA samples. Further validation of the DEGs, especially the novel DEGs, in future studies is warranted.

## Supporting information

S1 Fig Graphical summaries of identified genes within each treatment groups of both time points. (A) Number of genes detected in each of the four treatment groups. (B) The number of expresed genes with different expression levels against the range of PKPKM values. (C) The mean log10 transformed values of FPKM in four pooled samples. (D) The distribution of log10 transformed values of FPKM in four pooled samples.
(TIF)

S2 Fig The relationship of the four pooled samples. (A) The result of PCA analysis. (B) The result of cluster anlaysis.
(TIF)

S1 Table A list of differentially expressed genes validated by ddPCR. (DOCX)

S2 Table Primers designed and used to validate gene expression by ddPCR. (DOCX)

S3 Table A summary of past filter, high quality, and mapped reads. (DOCX)

S4 Table Line 6_3_ differentially expressed genes in response to MDV challenge in contrast to the control counterparts. (XLSX)

S5 Table Line 7_2_ differentially expressed genes in response to MDV challenge in contrast to the control counterparts. (XLSX)

S6 Table Functional enrichment of 95 differentially expressed genes in common to line 6_3_ and 7_2_ birds in response to MDV challenge. (XLSX)

S7 Table Differentially expressed genes identified between the control groups of line 6_3_ and 7_2_ birds. (XLSX)

S8 Table Differentially expressed genes identified between MDV-challenged groups of line 6_3_ and 7_2_ birds. (XLSX)

S9 Table A list of genes reportedly dysregulated in expression in response to MDV challenge. (XLSX)
