# Supplementary figures and images for: Comparative transcriptomics of genetically divergent lines of chickens in response to Marek’s disease virus challenge at cytolytic phase
Source: PLoS One. 2017 Jun 7;12(6):e0178923. doi: 10.1371/journal.pone.0178923 (PMC5462384; doi:10.1371/journal.pone.0178923)

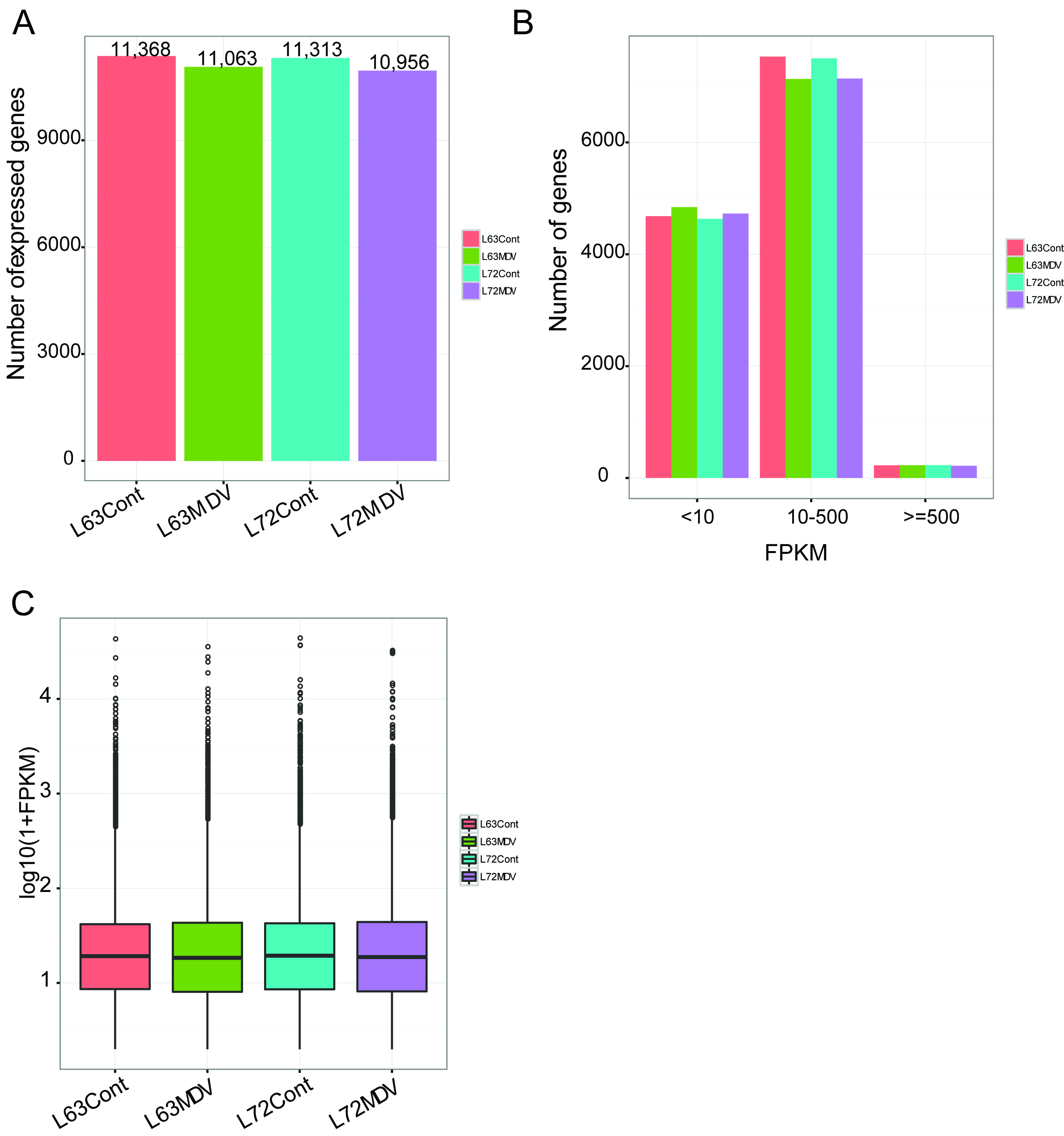

Supplement: S1 Fig — (A) Number of genes detected in each of the four treatment groups. (B) The number of expresed genes with different expression levels against the range of PKPKM values. (C) The mean log10 transformed values of FPKM in four pooled samples. (D) The distribution of log10 transformed values of FPKM in four pooled samples. (TIF) [file pone.0178923.s001.tif]

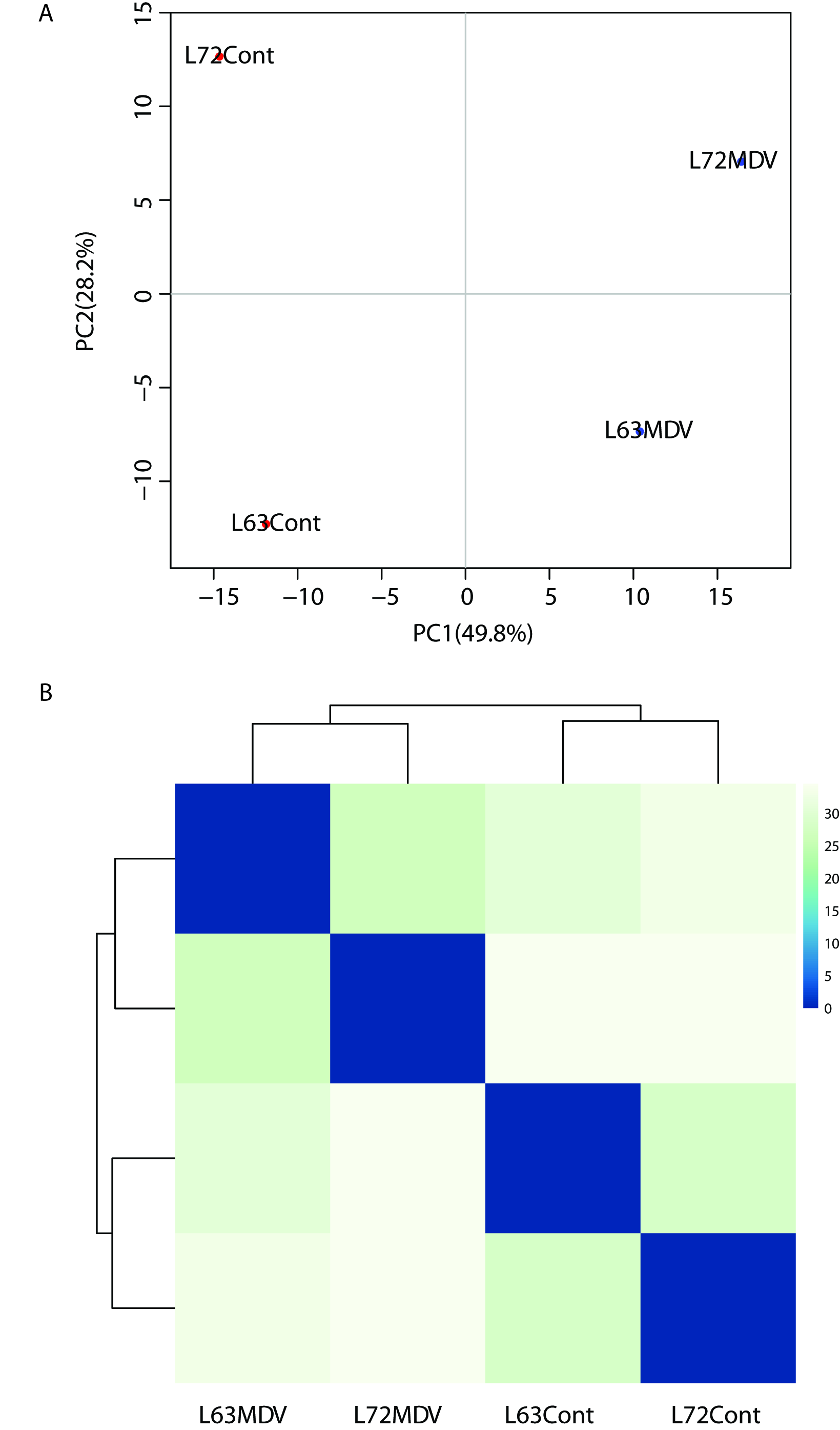

Supplement: S2 Fig — (A) The result of PCA analysis. (B) The result of cluster anlaysis. (TIF) [file pone.0178923.s002.tif]
