# Supplementary material for: Comparative transcriptomics of genetically divergent lines of chickens in response to Marek’s disease virus challenge at cytolytic phase
Source: PLoS One. 2017 Jun 7;12(6):e0178923. doi: 10.1371/journal.pone.0178923 (PMC5462384; doi:10.1371/journal.pone.0178923)
Supplement: S1 Table — (DOCX) [file pone.0178923.s003.DOCX]

**Table S1**. A list of differentially expressed genes validated by ddPCR

| **Gene** | **ddPCR** (Copies/µl) | | **RNA-seq** (FPKM^*^) | |
| --- | --- | --- | --- | --- |
|  | ***L6_3_Cont*** | ***L7_2_Cont*** | ***L6_3_Cont*** | ***L7_2_Cont*** |
| LECT2 | 545.6 | 39.96 | 2529.0 | 205.6 |
| FABP3 | 97.2 | 10.6 | 430.7 | 57.4 |
| RGS5 | 61.3 | 19.3 | 145.0 | 82.4 |
|  |  |  |  |  |
|  | ***L6_3_MDV*** | ***L7_2_MDV*** | ***L6_3_MDV*** | ***L7_2_MDV*** |
| PPT1 | 736.9 | 262.5 | 1369.0 | 890.3 |
| ATP6V0D2 | 22.4 | 149.1 | 84.2 | 987.0 |
| F13A1 | 64.2 | 86.0 | 223.7 | 677.0 |
|  |  |  |  |  |
|  | ***L6_3_MDV*** | ***L6_3_Cont*** | ***L6_3_MDV*** | ***L6_3_Cont*** |
| RGS5 | 18.7 | 61.3 | 62.8 | 145.0 |
| LECT2 | 289.6 | 545.6 | 854.3 | 2529.0 |
| ELN | 39.5 | 98.0 | 49.1 | 227.0 |
|  |  |  |  |  |
|  | ***L7_2_MDV*** | ***L7_2_Cont*** | ***L7_2_MDV*** | ***L7_2_Cont*** |
| ATP6V0D2 | 149.1 | 10.2 | 1002.7 | 95.1 |
| LECT2 | 351.5 | 289.7 | 1457.7 | 209.9 |
| PPT1 | 262.5 | 151.7 | 834.0 | 705.0 |

^*^Fragments Per Kilobase of transcript per Million
