# Supplementary material for: Comparative transcriptomics of genetically divergent lines of chickens in response to Marek’s disease virus challenge at cytolytic phase
Source: PLoS One. 2017 Jun 7;12(6):e0178923. doi: 10.1371/journal.pone.0178923 (PMC5462384; doi:10.1371/journal.pone.0178923)
Supplement: S2 Table — (DOCX) [file pone.0178923.s004.DOCX]

**Table S2.** Primers designed and used to validate gene expression by ddPCR

| **Gene** | **Primer (5’- 3’)** | **Amplicon Size (bp)** | **Annealing Tm (℃)** |
| --- | --- | --- | --- |
| *LECT2* | F: ATCTCTGGGTCAGGTTACTG | 188 | 58 |
|  | R: GGTATAGGTCTGAGGAGATGAG |  |  |
| *FABP3* | F: CCCATAGCACCTTCAAGAAC | 105 | 58 |
|  | R: CATCTAGCTTGACCAAGGAC |  |  |
| *RGS5* | F: GATCTACGAGGAGTTCATCCAG | 143 | 58 |
|  | R: CCATCAGAGCAAAGATCCTC |  |  |
| *PPT1* | F: GACTGGAATTCACCTGGATG | 112 | 58 |
|  | R: GAGACCAGGAGTAGATGTAGG |  |  |
| *F13A1* | F: CCTACATCCCGATCCTCATAG | 84 | 58 |
|  | R: GAATGGAGTTGTTCTCCCTG |  |  |
| *ATP6V0D2* | F: CAAGAATCCAGCAGAGAGAC | 133 | 58 |
|  | R: GTCTGCACTAGTCAAGATCC |  |  |
| *ELN* | F: GAAATACTGTGGGAGGAAGC | 97 | 58 |
|  | R: GGTGGGGATTTACATTCTGG |  |  |
