# Supplementary material for: Comparative transcriptomics of genetically divergent lines of chickens in response to Marek’s disease virus challenge at cytolytic phase
Source: PLoS One. 2017 Jun 7;12(6):e0178923. doi: 10.1371/journal.pone.0178923 (PMC5462384; doi:10.1371/journal.pone.0178923)
Supplement: S3 Table — (DOCX) [file pone.0178923.s005.docx]

**Table S3.** A summary of past filter, high quality and mapped reads

| **Treatment group** | **Past filter reads** | **High quality**  **reads** | **Mapped**  **reads** | **Map**  **rate** |
| --- | --- | --- | --- | --- |
| L6_3_ Control | 32,444,975 | 26,580,374 | 22,751,486 | 85.60% |
| L7_2_ Control | 33,012,628 | 27,098,303 | 23,603,093 | 87.10% |
| L6_3_ MDV | 40,358,481 | 33,239,329 | 29,195,651 | 87.83% |
| L7_2_ MDV | 28,286,638 | 23,269,825 | 20,500,235 | 88.10% |
